# Assessing progression in Bipolar Disorder: a staging model tested in a sample over ten years of observation

**DOI:** 10.1192/j.eurpsy.2023.1205

**Published:** 2023-07-19

**Authors:** N. Girone, M. Macellaro, L. Cremaschi, M. Bosi, B. M. Cesana, F. Ambrogi, B. Dell’Osso

**Affiliations:** 1 Department of Mental Health, Department of Biomedical and Clinical Sciences, University of Milan, Luigi Sacco Hospital; 2 Department of Clinical Sciences and Community Health, Unit of Medical Statistics, Biometrics and Bioinformatics, Giulio A. Maccacaro”, Faculty of Medicine and Surgery, University of Milan, Milan, Italy; 3 Department of Psychiatry and Behavioral Sciences, Bipolar Disorders Clinic, Stanford University, Stanford, CA, United States; 4 Center for Neurotechnology and Brain Therapeutic, University of Milan, Centro “Aldo Ravelli”, Milan, Italy

## Abstract

**Introduction:**

The longitudinal course of bipolar disorder (BD) is related to an active process of neuroprogression, associated with brain changes and functional impairment (Berk et al., *Bipolar Disord* 2014; 16(5):471-7). Several clinical factors may influence illness trajectories, including the number of episodes and hospitalizations, the presence of comorbidities, stressful life events and familiarity for psychiatric disorders (Post. *Braz J Psychiatry* 2020;42(5):552-557). Trying to better define such progression, several authors conceptualized different staging models for BD, each one emphasizing different aspects of illness.

**Objectives:**

In the present study, we focused on the Kupka & Hilleghers staging model, owing to its favorable ratio between the number of classes and transitions (Kupka & Hilleghers. *Tijdschr Psychiatr* 2012; 54(11):949-956). The aim was to investigate the transition of a sample of 100 BD patients through the different stages of illness across 10 years of observation, analyzing the potential role of clinical variables on the risk of illness progression.

**Methods:**

Clinical stages of 100 BD patients (53 BDI and 47 BDII) were retrospectively assessed according to the model proposed by Kupka & Hilleghers at four time points: T0 (2010), T1 (2015), T2 (2018) and T3 (2020, at inclusion). Multistate Model using the mstate package in R and Markov model with stratified hazards were used for statistical analysis, to assess transition intensities across illness stages and the potential role of clinical variables on the risk of progression.

**Results:**

A significant stage progression emerged during the observation period (Figure 1). More in detail, high hazard of transition from stage 2 to stage 3 was observed (Figure 2). A significant effect on the transition rate from 3 to 4 was found for higher number of affective episodes lifetime (> 3 episodes) (p=0.03) and for elevated predominant polarity (p=0.01). Overall, the average time subjects spent in stage 0 was 30.8 years and for stage 1 was 0.78 years. After BD onset, patients spent an average of 0.78 years in stage 2, 6.21 years in stage 3 and 2.23 in stage 4.

**Image:**

**Figure:**
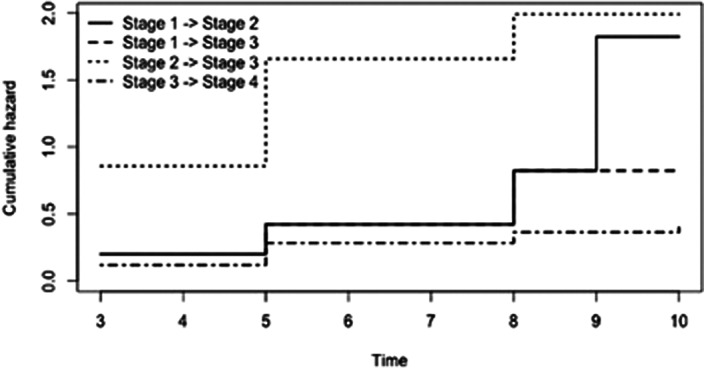

**Image 2:**

**Figure:**
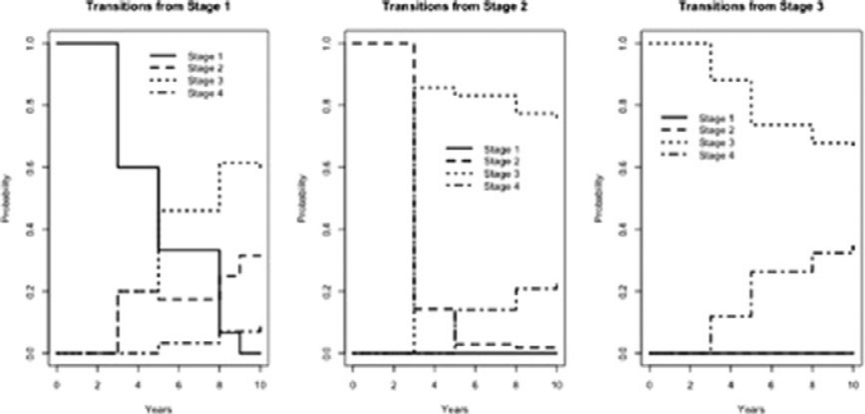

**Conclusions:**

Present preliminary results confirm the progressive nature of the disorder. An increased risk of transition across stages emerged for patients with higher number of episodes lifetime and with elevated predominant polarity, confirming the need of improving timing and accuracy of diagnosis and therapeutic interventions. Further studies are warranted with the aim of define a universal staging model for BD.

**Disclosure of Interest:**

None Declared

